# Origin and evolution of candidate mental retardation genes on the human X chromosome (MRX)

**DOI:** 10.1186/1471-2164-9-65

**Published:** 2008-02-05

**Authors:** Margaret L Delbridge, Daniel A McMillan, Ruth J Doherty, Janine E Deakin, Jennifer A Marshall Graves

**Affiliations:** 1 Comparative Genomics Group, Research School of Biological Sciences, The Australian National University, Canberra, ACT 2601, Australia

## Abstract

**Background:**

The human X chromosome has a biased gene content. One group of genes that is over-represented on the human X are those expressed in the brain, explaining the large number of sex-linked mental retardation (MRX) syndromes.

**Results:**

To determine if MRX genes were recruited to the X, or whether their brain-specific functions were acquired after relocation to the mammalian X chromosome, we examined the location and expression of their orthologues in marsupials, which diverged from human approximately 180 million years ago. We isolated and mapped nine tammar wallaby MRX homologues, finding that six were located on the tammar wallaby X (which represents the ancient conserved mammal X) and three on chromosome 5, representing the recently added region of the human X chromosome. The location of MRX genes within the same synteny groups in human and wallaby does not support the hypothesis that genes with an important function in the brain were recruited in multiple independent events from autosomes to the mammalian X chromosome. Most of the tammar wallaby MRX homologues were more widely expressed in tammar wallaby than in human. Only one, the tammar wallaby *ARX *homologue (located on tammar chromosome 5p), has a restricted expression pattern comparable to its pattern in human. The retention of the brain-specific expression of *ARX *over 180 million years suggests that this gene plays a fundamental role in mammalian brain development and function.

**Conclusion:**

Our results suggest all the genes in this study may have originally had more general functions that became more specialised and important in brain function during evolution of humans and other placental mammals.

## Background

For more than a century, it has been obvious that mental retardation is far more frequent in human males than females, and there have been many claims over the years that inherited mental retardation (MR) syndromes are concentrated on the X chromosome [1,2]. Several well studied X-borne genes have an effect on general cognitive abilities, including *FMR1 *(fragile X syndrome) and *FMR2 *(FRAXE mental retardation), as well as a large number of X-linked mental retardation (MRX) syndromes that have been mapped but not cloned and characterised [3]. Attempts to prove that MRX genes are located disproportionately on the X have suffered from experimental ascertainment bias, since recessive MRX genes are more easily recognised in hemizygous males. However, one analysis of the OMIM database corrected for any ascertainment bias by comparing the proportions of X-linked phenotypes of MR syndromes to that of other abnormalities [4]. Another analysis tested whether the over-representation of MRX syndromes would disappear if as yet unmapped MR genes were evenly distributed across the genome [5]. Both analyses concluded that mental retardation syndromes are significantly concentrated on the human X chromosome. Other gene functions have also been found to be unequally represented on the human X chromosome. For example, early spermatogonial genes, sex and reproduction related (SRR) and prostate-specific genes are over-represented on the X in humans and mice, whereas there is a paucity of genes involved in late spermatogenesis [6-10].

How this over-representation occurred has been the subject of much speculation, and several alternative hypotheses have been considered. One possibility is that brain function genes, or copies of them, were moved one by one to the X from autosomes, another is that the over-representation of genes on the X was the result of the translocation of a larger autosomal region already rich in brain function genes to the X. Alternatively, it has been suggested that the gene content of the X chromosome has remained unchanged since its autosomal origin, but some X-borne genes have acquired new functions in brain development.

These hypotheses must be tested within the framework of human sex chromosome evolution. Mammals have an XX female: XY male chromosomal sex determination system, in which a gene on the Y chromosome controls male development. Homology between the X and Y chromosomes supports the hypothesis that the mammalian X and Y chromosomes differentiated from a homologous autosomal pair (the proto-X and proto-Y) when one member acquired a male-determining allele. Suppression of recombination between the X and Y, loss of active genes and accumulation of mutations on the Y resulted in the progressive degradation of the Y such that most genes on the X chromosome have no homologue on the Y chromosome.

The X chromosome gene content of different eutherian mammals is highly conserved, perhaps because of the chromosome-wide dosage compensation mechanism that ensures equal expression of X-linked genes in males and females. Mapping human X-borne genes in distantly related mammals [11] showed that only part of the X chromosome is conserved between the eutherians ("placental" mammals) and marsupials (which diverged from eutherians 180 million years ago). The long arm and pericentric region of the human X is equivalent to the marsupial X and is therefore at least 180 million years old. This region of the human X chromosome is known as the conserved region of the X (XCR). However, genes on the short arm of the human X are autosomal in marsupials, implying the addition of a region to the sex chromosomes between 130 and 80 million years ago. This region of the human X chromosome is known as the recently added region (XAR) and it is exposed to the same forces that shaped the ancient region. These building blocks of the human X chromosome are evident as two major autosomal regions in chickens [12]. The different evolutionary histories of the ancient and added regions of the human X can be exploited to test hypotheses of how and why mental retardation genes came to accumulate on the X chromosome.

The presence of the X chromosome as two copies in females, but only a single copy in males may explain the accumulation of certain classes of genes on the X chromosome that confer an advantage to males but are neutral, or even deleterious, in females [13]. Alternative hypotheses to explain the accumulation of MRX genes on the human X may be tested by observing the location and expression of their marsupial orthologues. Mapping will show whether the MRX genes were part of ancient gene blocks, or were recruited to the human X chromosome from autosomes as independent events. If they were part of ancient gene blocks, it would be expected that MRX genes from the conserved region of the human X would also be located on the X chromosome in the tammar wallaby, and that MRX genes from the recently added region of the human X would be found on the short arm of chromosome 5 in the tammar wallaby. If MRX genes were recruited from autosomes to the X chromosome as independent events, their homologues would be likely to be distributed across the genome in the tammar wallaby. Examination of the expression profiles of marsupial orthologues of MRX genes on the conserved and the recently added regions of the human X chromosome will test the whether the function of these genes has changed due to their location on the X. If genes on both the tammar wallaby X chromosome and chromosome 5p have brain-restricted expression patterns like their human counterparts, then this would support the hypothesis that recently added region of the human X chromosome was rich in brain function genes prior to its translocation to the ancient X, and their function was retained after relocation to the X. If genes located on the wallaby X have brain-specific expression like their human counterparts, but genes on the wallaby 5p have a different expression pattern, this supports the hypothesis that the function of these genes has changed in the eutherian lineage upon relocation to the X chromosome.

To test this hypothesis we studied a number of MRX genes, and candidate MRX genes involved in brain function distributed along the human X chromosome.

## Results

### Choice of human genes for this study

We chose genes that are more or less evenly distributed along the human X chromosome and are involved in a number of biological pathways or neuronal processes (Table 1). They include genes such as *OPHN1*, *ARHGEF6 *and *TSPAN7*, which play a role in the formation and dismantling of the actin cytoskeleton to control growth of neurons, and genes such as *RSK2*, *JARID1C *and probably *RSK4*, which play a role in the control of gene expression through modulation of chromatin structure. Other genes are likely to regulate transcription and translation, such as *ARX*, *FMR1 *and *FMR2*, or encode receptor proteins, such as *SREB3 *and *AGTR2*. Most of these genes are human MRX genes, specific mutations in which cause mental retardation. Mutations that cause mental retardation have not yet been found in the *RSK4 *and *SREB3 *genes, but *RSK4 *is strong candidate MRX gene because of its similarity to *RSK2*, a known MRX gene, and *SREB3 *because of its brain-specific expression pattern. Some of the MRX genes are ubiquitously expressed and mutations within these genes cause cellular defects in many tissues, as well as predominantly affecting the brain because of its higher sensitivity [5]. Regardless of the role these genes play in the brain of eutherian mammals, many are expressed in the brain more strongly than in other tissues, and many have a period during development when expression is restricted to the brain or nervous system (Table 1).

**Table 1:** Summary of the location, function and expression patterns of the human genes in this study.

Locus symbol	Gene name	Human location	Proposed function	Expression in human and/or mouse	reference
*RSK2 (RPS6KA3)*	ribosomal S6 kinase 2	Xp22.2 (XAR)	Serine threonine protein kinase that phosphorylates CREB and regulates histone H3 acetyltransferase, to play a role in long term memory	Expressed widely, but only at low levels in foetal and adult tissues; is concentrated in the adult brain, skeletal muscle and heart	[24–26]
*ARX*	*Aristaless*-related homeobox gene	Xp22 (XAR)	Transcription factor with a role in neuronal proliferation and differentiation	Restricted to embryonic brain in human, but also found in mouse testis. Widespread strong expression in the adult heart, skeletal muscle, kidney and liver, no expression in the brain.	[27–30]
*TSPAN7 (TM4SF2)*	tetraspanin 7	Xp11.4 (XAR)	Modulates integrin-mediated signalling, to play a role in neurite outgrowth and synapse formation	Ubiquitous in foetus and adult	[31]
*SREB3 (GPR173)*	Super conserved receptor expressed in the brain	Xp11.22 (XCR)	G- protein coupled receptor, role in MRX unknown	Restricted to brain and ovary with low levels detectable in the small intestine	[32, 33]
*JARID1C (SMCX)*	Jumonji AT-rich interactive domain 1C	Xp11.22 (XCR)	Role in chromatin remodelling by regulating transcription	Widely expressed at different levels in different tissues. Strongest in adult brain and skeletal muscle.	[34]
*OPHN1*	Oligophrenin 1	Xq12 (XCR)	Negatively regulates Rho-GTPase activity to affect formation and dismantling of the actin cytoskeleton and control of neurite growth	Ubiquitous in foetus, restricted to central and peripheral neuronal tissues in the adult	[35–37]
*RSK4 (RPS6KA6)*	ribosomal S6 kinase 4	Xq21 (XCR)	Serine threonine kinase with very high homology to *RSK2*, suggesting a role in normal cell proliferation and neuron differentiation	Widespread weak expression in adult, concentrated in the adult brain and kidney	[24, 38, 39]
*AGTR2*	angiotensin II receptor, type 2	Xq22 (XCR)	Receptor protein with a role in central nervous system or cardiovascular function	Highly expressed in all developing foetal tissues, but adult expression only in brain, adrenal medulla, and atretic ovary	[40, 41]
*ARHGEF6*	rho guanine nucleotide exchange factor 6	Xq26 (XCR)	activates Rho-GTPase activity to affect formation and dismantling of the actin cytoskeleton and control of neurite growth	Ubiquitous	[35, 42, 43]
*FMR1*	fragile X mental retardation 1 gene	Xq27.3 (XCR)	RNA-binding protein with roles in synaptic maturation and function, and in germ cell proliferation	Widely expressed in most adult and foetal tissues, highest in brain and the testis	[44–46]
*FMR2*	fragile site mental retardation 2 gene	Xq28 (XCR)	Nuclear transcriptional regulator with a role in learning and memory	Restricted to placenta and adult brain.	[47, 48].

### Isolation and mapping of tammar wallaby MRX gene orthologues

To isolate BAC clones containing orthologues of human MRX genes from the tammar wallaby BAC library, homologous human, mouse and Brazilian gray short-tailed opossum (*Monodelphis domestica*) sequences available from the public databases were compared with the tammar wallaby trace archives to identify highly conserved gene sequences. The availability of tammar wallaby sequences in the trace archive allowed the design of tammar wallaby primer pairs to amplify the *RSK4*, *AGTR2 *and *ARHGEF6 *genes from female tammar wallaby genomic DNA (Table 2). Primer pairs designed from available opossum sequence were used to amplify the *RSK2*, *ARX*, *OPHN1*, and *FMR2 *genes from female tammar wallaby genomic DNA (Table 2). PCR products of the expected size (Table 2) were cloned and sequenced to verify their identity and were used separately to identify positive BAC clones from the tammar wallaby BAC libraries. To identify BAC clones containing the *FMR1 *and *TSPAN7 *gene, 40bp overgos designed from opossum (*FMR1) *or tammar wallaby (*TSPAN7) *sequence were used to screen the tammar wallaby BAC library.

**Table 2:** Summary of primers used for library screening and expression analysis. BAC clone addresses are also given for each BAC clone identified and used in the localisation of the MRX genes.

Gene	Primer Pair	Use	Sequence	BAC clone	Product size genomic/cDNA	Anneal°C
RSK2	MdRSK2 (17)F1 MdRSK2 (17)R1 MeRSK2 (13)F1 MeRSK2 (20)R1	BAC isolation expression	F1: 5'-TGAAAGGAGGTGAACTACTGGA-3' R1: 5'-ACCCCTTGTGCATGAAGATA-3' F1: 5'-AGCCTGCAACAGGTAGACCT-3' R1: 5'-TTTGCAGTGTCTGAAACGGA-3'	VIA 73C9	118 bp 1.0 kb	50 55
*ARX*	MdARX F5 MdARX R5	BAC isolation/expression	F5: 5'-AGCTCTGAGGCTGAAAGCAA-3' R5: 5'-TTTGATTAGCGATGGTGGTG-3'	VIA 37C12	150 bp	57
*TSPAN7*	MeTSPAN7 OVa MeTSPAN7 OVb MdTM4SF2 F2 MdTM4SF2 R2	BAC isolation expression	5'-TGCCATGTTCTTGTCCCTGGTGTT-3' 5'-CACCAGCTCAGCCAAGAACACCAG-3' F2: 5'-GAGCTGCTGTGGTGTTCAGA-3' R2: 5'-CAAACCTTTTGATTGACTTTAGTGG-3'	AGI 383N14	40 bp 150 bp	n/a 58
*SREB3*	MdSREB3 F2 MdSREB3 R2	BAC isolation/expression	F2: 5'-CTTGCACAAGGCTCCCTACT-3' R2: 5'-TCATGCGTTTGGCATAGAAG-3'	AGI 63D15*	270 bp	52
*JARID1C*	MeSMCX F1 MeSMCX R1	BAC isolation/expression	F1: 5'-CGTCTGAGGAGGAGCCATAG-3' R1: 5'-CCTTGAAGGAGTCAGCCATC-3'	AGI 63D15*	250 bp	57
*OPHN1*	MdOPHN1 F2 MdOPHN1 R2	BAC isolation/expression	F2: 5'-CACAGCCGAGAGAACCTGAT-3' R2: 5'-GGATTTCCACGACGATGTTC-3'	AGI 62P8	118 bp	59
*RSK4*	MeRSK4 F2 MeRSK4 R2 MeRSK4 (13)F1 MeRSK4 (20)R1	BAC isolation expression	F2: 5'-CATTCAAACCTGCATCTGGA-3' R2: 5'-TCGCTTGCAAACAGAATAGG F1: 5'-AACCTGCATCTGGAAAACCA-3' R1: 5'-TTTGCTGCATCTGAAACAGT-3'	AGI 88I19	650 bp 1.0 kb	60 55
*FMR1*	MdFMR1 OVa MdFMR1 OVb MeFMR1 F1 MeFMR1 R1	BAC isolation expression	5'-TGGTGCTAATAATCAGCAAGCTAG-3' 5'-GACTCCAGGTACTTTTCTAGCTTG-3' F1: 5'-GACAGCATCACCAATGCAAC-3' R1: 5'-CGCTGACGATTATCTGTTCG-3'	AGI 77O12	40 bp 450 bp	n/a 59
*FMR2*	MdFMR2 F1 MdFMR2 R1 MeFMR2 F2 MeFMR2 R2	BAC isolation expression	F1: 5'-CTGCTAGCCATGTCAACATCA-3' R1: 5'-TCAGTCTGTCTGCCATGTCC-3' F2: 5'-AGAGCCTCCTTCAACCAACA-3' R2: 5'-CCTGTTCTGGGTGTTCCAGT-3'	AGI 39E1	80 bp 450 bp	59 60
*AGTR2*	MeAGTR2 F1 MeAGTR2 R1	BAC isolation/expression	F1: 5'-TACCAAGCCGTTGTCTACCC-3' R1: 5'-CTGCGGAGCTGAGAAACTCT-3'	AGI 91P8	600 bp/650 bp	59
*ARHGEF6*	MeARHGEF6 F1 MeARHGEF6 R1	BAC isolation/expression	F1: 5'-TTGGGCAACTTTGAGGAAGT-3' R1: 5'-CATTCACAGCTGAGGGATGA-3'	AGI 139J23	2.0 kb/170 bp	57

* Delbridge et al, in preparation.

To verify that the positive BAC clones identified by library screening contained the correct gene, BAC DNA was isolated, and used as the template for PCR amplification of each gene using the primer pairs used to generate the original PCR fragment for library screening. Tammar wallaby trace sequences were used to design primers to amplify an *FMR1 *gene fragment from the BAC clones. PCR products generated from the BAC clones were sequenced to verify that the BAC clones contained the correct gene sequence. The *TSPAN7 *positive BAC was verified by sequencing using the overgos as primers in the sequencing reaction. This allowed the identification of tammar wallaby BAC clones containing sequences homologous to nine MRX genes from the human X chromosome. The possibility that these were intronless retroposed copies was eliminated by the amplification and sequencing of intron-containing PCR products from each BAC clone, demonstrating that the sequence of these products was identical to that of the original probes.

**Figure 1:**
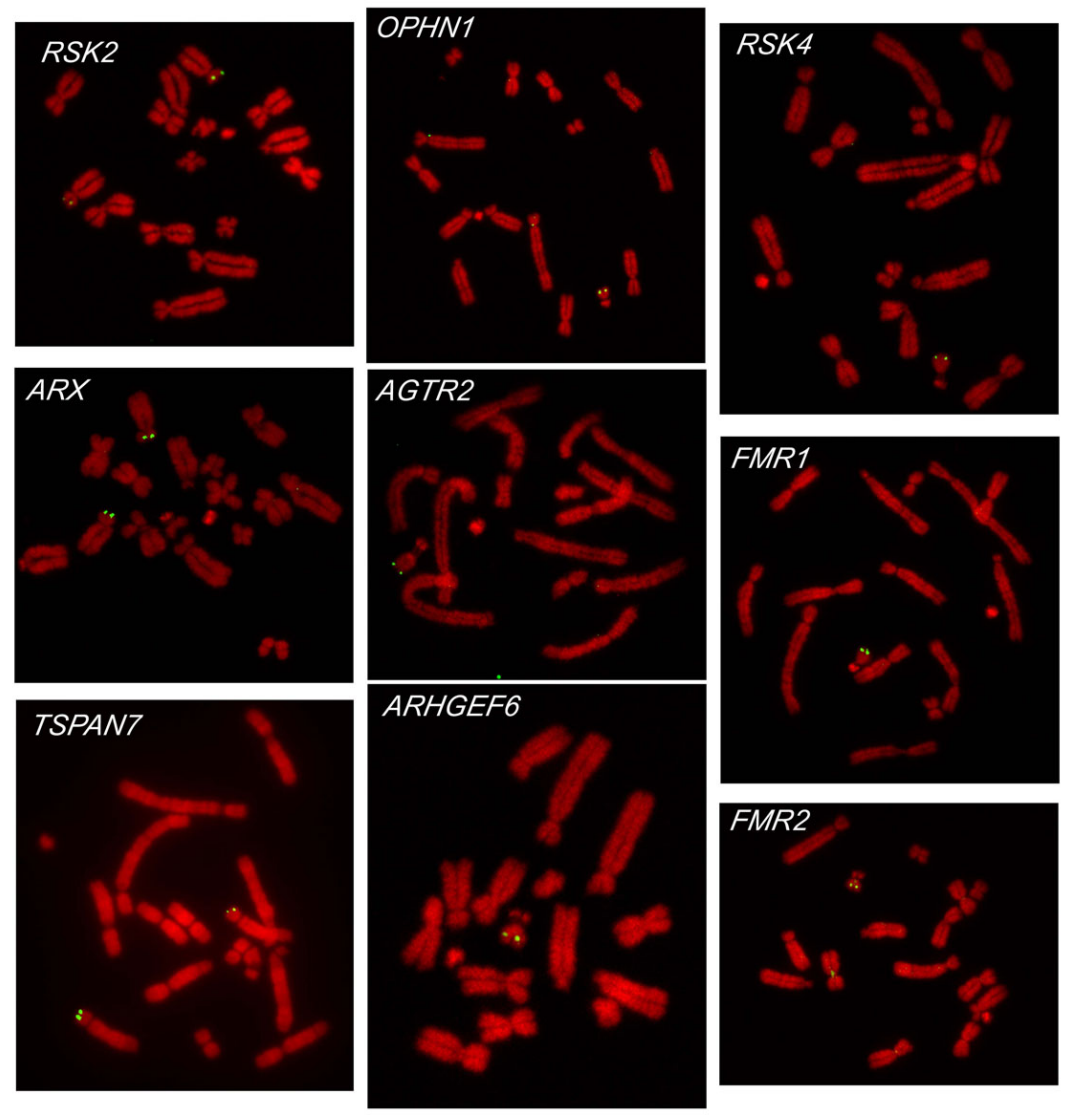
**Localisation of tammar wallaby MRX homologues**. BACs containing tammar wallaby MRX homologues hybridised to male metaphase chromosome spreads. Signals are visualised in green, and chromosomes are counterstained with DAPI (4',6-diamidino-2-phenylindole) and visualised in red.

Fluorescence *in situ *hybridisation (FISH) was used to map the verified BAC clones onto tammar wallaby metaphase chromosome spreads. BAC clones containing the tammar wallaby homologues of human genes located in the conserved region of the human X chromosome (*OPHN1*, *RSK4*, *AGTR2*, *ARHGEF6*, *FMR1 *and *FMR2*) hybridised to the long arm of the tammar wallaby X chromosome (Figure 1). BAC clones containing the tammar wallaby homologues of human genes located in the recently added region of the human X chromosome (*RSK2*, *ARX*, and *TSPAN7*) hybridised to the short arm of tammar wallaby chromosome 5.

These results imply that these tammar wallaby MRX homologues are part of the previously defined conserved and recently added regions of the human X chromosome, which are represented by the X chromosome and the short arm of chromosome 5 in the tammar wallaby [11]. They are clearly parts of ancient gene blocks, not the result of independent or more recent transposition events to the mammalian X chromosome, which is consistent with what we already know about the evolution of the eutherian X chromosome [14].

### Expression of MRX homologues in the tammar wallaby

We examined the expression profiles of all the tammar wallaby orthologues of human MRX and candidate MRX genes. This included XAR genes *RSK2*, *ARX*, and *TSPAN7 *from the short arm of tammar wallaby chromosome 5, and XCR genes *OPHN1*, *RSK4*, *AGTR2*, *ARHGEF6*, *FMR1 *and *FMR2 *from the tammar wallaby X chromosome. Two other genes previously localised to the tammar wallaby X chromosome, *SREB3 *and *JARID1C *(Delbridge in preparation), were also included in this analysis.

**Figure 2:**
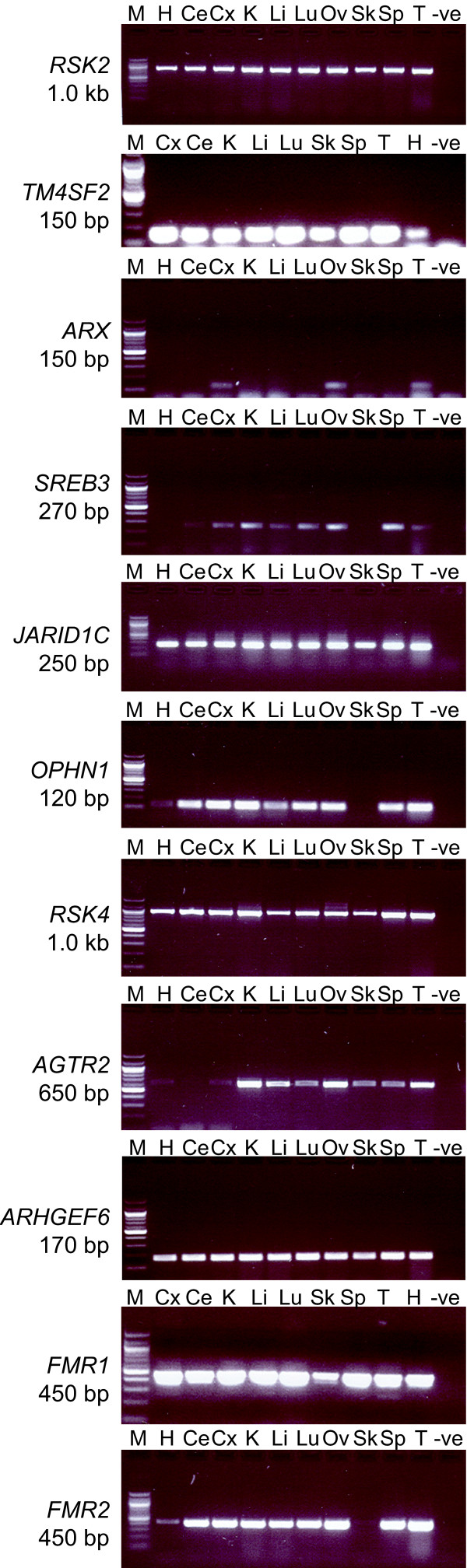
**Expression profiles of tammar wallaby MRX homologues**. Ce: cerebellum, Cx: cortex, H: heart, K: kidney, Li: liver, Lu: lung, Ov: ovary, Sk: skeletal muscle, Sp: spleen, T: testis, -ve: negative control. M: 1.0 kb marker (Roche, Australia).

We found that *RSK2, TSPAN7, SREB3, JARID1C, OPHN1, ARHGEF6, FMR1*, *FMR2, RSK4*, and *AGTR2 *transcripts could be amplified by RT-PCR from RNA extracted from all adult tissues tested (Figure 2). For some genes the amount of RT-PCR product varied in different tissues. Tammar wallaby *SREB3*, *RSK2 *and *AGTR2 *were only weakly expressed in the brain and some other tissues (Figure 2). Tammar wallaby *OPHN1*, *FMR2 *and *SREB3 *were expressed at a lower level in muscular tissues such as the heart and the skeletal muscle, whereas RT-PCR products produced by amplifying other genes from the same samples gave bands of similar intensity in all tissues. Despite these differences in the levels of expression the presence of an RT-PCR product in all tissues implies that these genes are widely expressed in tammar wallaby, in contrast to their tissue-specific expression in human and mouse (Table 3).

**Table 3:** Tammar wallaby localisation and expression data compared to human. Ad: adrenal medulla, B: brain, H: heart, K: kidney, Ov: ovary, Pl: placenta, Sk: skeletal muscle, Sp: spleen, Te: testis.

Gene	Human/mouse expression	Tammar wallaby location	Tammar wallaby expression
*RSK2*	ubiquitous	5p	ubiquitous
*ARX*	embryonic B, Te, adult H, Sk, Li, K	5p	B, Te, Ov
*TSPAN7*	ubiquitous	5p	ubiquitous
*SREB3*	B, Ov	Xq	ubiquitous
*JARID1C*	ubiquitous	Xq	ubiquitous
*OPHN1*	B	Xq	ubiquitous
*RSK4*	ubiquitous	Xq	ubiquitous
*AGTR2*	B, Ad, Ov	Xq	ubiquitous
*ARHGEF6*	ubiquitous	Xq	ubiquitous
*FMR1*	ubiquitous	Xq	ubiquitous
*FMR2*	B, Pl	Xq	ubiquitous

In contrast, *ARX *expression was restricted to the brain (cortex), testis and ovary tissues in the adult tammar wallaby (Figure 2). Two pairs of primers were used to amplify RT-PCR products within exon 5 (Figure 2) and spanning exons 2 to 5 (results not shown), and include most of the coding region of *ARX*. Both primer pairs amplified only a single product from cortex, testis and ovary tissues from both tammar wallaby adult and pouch young (day 97 after birth), indicating that restricted *ARX *expression occurs at both stages. Our experiments would not detect the different isoforms of *ARX *that have been proposed to result from changes in the 3' untranslated region rather than the coding region [15]. The tammar wallaby *ARX *expression profile more closely reflects the brain and testis specific expression pattern of *ARX *in human and mouse embryos, rather than the more widespread expression profile observed in human adults. Unlike its expression profile in eutherian mammals, *ARX *was expressed in the tammar wallaby ovary.

## Discussion

### Did human MRX genes originate from autosomal sites?

One hypothesis to explain the over-representation of brain-expressed genes on the human X is that brain-specific genes were transferred to the X from autosomal sites. We tested this hypothesis by determining the chromosomal location of the tammar wallaby orthologues of nine X linked human MRX genes. Tammar wallaby orthologues of XCR genes on the long arm of the human X chromosome *(OPHN1*, *RSK4*, *AGTR2*, *ARHGEF6, FMR1*, and *FMR2) *were located on the X chromosome in the tammar wallaby. Two other genes (*SREB3 *and *JARID1C*) lie close to the centromere of the short arm of the human X. This region is part of the ancient therian X, but its separate location in chickens and fish mean that it represents a separate evolutionary block [16]. Tammar wallaby orthologues of these genes also lie on the tammar wallaby X (Delbridge in preparation). Thus all the MRX genes in the conserved region of the human X were part of the original therian X chromosome.

Human *RSK2*, *ARX *and *TSPAN7 *are located on the short arm of the human X chromosome in a region that is autosomal in marsupials, and therefore represents a recent addition to the eutherian X. As expected, tammar wallaby orthologues of these XAR genes were located on the short arm of chromosome 5, mapping among other genes from this region. This confirms that these MRX genes were part of the recently added region that was transferred to the eutherian X chromosome following the divergence of eutherians from marsupials.

We conclude, therefore, that these eleven human MRX genes were part of two previously known conserved blocks of genes, the XAR and XCR, which make up the human X chromosome. None were transferred to the human X chromosome from other locations as independent acquisitions, or as different evolutionary blocks. This is also consistent with the available mapping data from another marsupial, the Brazilian short-tailed grey opossum (*Monodelphis domestica*), in which XCR genes lie on the X and XAR genes on opossum chromosome 4 [17]. In the more distantly related platypus (*Ornithorhynchus anatinus*), XCR genes lie on chromosome 6 [18], and XAR genes on two smaller chromosomes (Veyrunes, in preparation).

### Did human MRX genes originate from genes with more general function?

Given that MRX genes are part of ancient conserved blocks, their over-representation on the X could be due either to an over-representation on the autosomal blocks that evolved into sex chromosomes, or the acquisition of more specialised function by genes with generalised expression. We distinguished between these hypotheses by examining the expression patterns of the MRX genes in the tammar wallaby. The hypothesis that these genes had brain-specific function in the ancestral mammal predicts that their expression will be the same in marsupials as eutherians. The hypothesis that genes with a more general function acquired brain-specific functions predicts that, at least for a few genes, expression patterns in marsupials and eutherians will be different. Moreover, genes more recently recruited to the X (those mapping to tammar wallaby 5p) might show less specialization, and fewer differences.

We obtained expression profiles from the homologues of the eleven genes in the tammar wallaby (Figure 2). Ten of these (*SREB3*, *JARID1C*, *OPHN1*, *RSK4*, *AGTR2*, *ARHGEF6, FMR1*, *FMR2*, *RSK2*, and *TSPAN7*) were widely expressed in tammar wallaby; RT-PCR products were detected in most of the tissue samples tested, including the brain (Table 3). Of the human XCR genes, four (*JARID1C*, *RSK4*, *ARHGEF6 *and *FMR1*) are also widely expressed in humans, so no difference in expression was detected. However, the wide expression of tammar wallaby orthologues of four human XCR genes (*SREB3*, *OPHN1*, *AGTR2 *and *FMR2) *contrasts with a more restricted expression pattern in human, suggesting that the brain functions, at least of these genes, were recently acquired in the therian lineage.

Tammar wallaby orthologues of two human Xp genes lying in the added region (*RSK2 *and *TSPAN7) *were also widely expressed, as they are in humans. The wide expression of these MRX genes from the added region of the human X suggests that they acquired a specific function more recently, only in the human brain.

The *ARX *gene in the added region of the human X is particularly interesting because it alone has a restricted expression pattern (cortex of the brain, testis and ovary) in the tammar wallaby. This reflects the restricted expression pattern in eutherian embryos, but differs from the wide expression in adults. Thus, *ARX *expression is restricted to the developing brain and reproductive tissues in all therian mammals. This pattern of expression appears to have been retained in the adult tammar wallaby, but has been relaxed to include expression in other tissues in adult human and mouse. *ARX *is therefore an example of a gene that has always played a critical role in development of the therian brain. Since *ARX *lies on tammar wallaby chromosome 5p, this ancient brain-specific function was not a consequence of its location on the X chromosome.

Expression patterns of MRX homologues have not yet been examined in a wide range of tissue types from the platypus or the chicken so we cannot deduce expression patterns of the MRX genes in an ancestral tetrapod. The expression patterns of *OPHN1*, *ARHGEF6 *and *FMR1 *have been examined in detail in the chicken brain [19] and found to be similar in the ancient regions of the mouse and chicken brain such as the cerebellum, but very different in the younger regions of the brain such as the telencephalon. This suggested that at least these three X-borne genes independently evolved specialised functions in the brain following the divergence of birds and mammals [19]. We found that these three genes were expressed widely in adult tammar wallaby tissues, including the brain, but since we did not examine expression in the corresponding different regions of the brain, we cannot tell whether this change in expression occurred before or after the marsupial-eutherian divergence.

## Conclusion

In conclusion, our results are not consistent with the hypothesis that the over-representation on the X chromosome of genes with a function in brain resulted from relocation or copying of brain function genes from autosomes, either independently or as transposed blocks of independent origin. It remains possible that the over-representation of these genes was a property of the ancient autosomal regions that became the sex chromosomes. At least some brain-specific genes on the human X chromosome, such as *ARX*, already had a brain-specific function when they were located on the autosomal proto-X chromosome. However, we present four examples of the narrowing of the expression patterns of human XCR genes. This suggests that the acquisition of brain-specific function by originally widely expressed genes is responsible for the over-representation of brain-specific genes on the human X chromosome.

## Methods

### PCR

PCR amplifications were carried out using 15 pmol of each primer (Geneworks, Adelaide, Australia), 2.0 mM each of dATP, dCTP, dGTP, dTTP (Roche, Sydney, Australia), and 0.625U *Taq *Polymerase in the recommended buffer containing 1.5 mM MgCl_2 _(Promega, Sydney Australia). Following an initial denaturation at 94°C for 2 minutes, cycling conditions were 35 cycles of 94°C for 30 seconds; 50–60°C annealing for 30 seconds; 72°C for 1 minute; with a final extension of 10 minutes at 72°C. Annealing temperatures are listed in Table 2. All PCR products were cloned into the TA TOPO Cloning Kit (Invitrogen, Australia) for sequencing and verification of their identity. Sequencing was done at the Australian Genomic Research Facility (AGRF), Brisbane, Australia.

### BAC libraries

BAC clones were isolated from either a male tammar wallaby (*Macropus eugenii*) BAC library (constructed at the Victorian Institute of Animal Sciences, VIA prefix, [20]) or the commercially available female tammar wallaby BAC library (Arizona Genomics Institute, AGI prefix).

### Library screening

Gene specific DNA probes generated by PCR (Table 2) were radioactively labelled using ^32^-P dATP or ^32^-P dCTP using the Megaprime DNA labelling system (Amersham, Australia). DNA probes were hybridised to BAC filters for 16 hours at 60°C in Church's buffer [21] and washed twice in 2×SSC/0.1% SDS at 60°C. DNA probes generated from mouse genomic DNA were hybridised and washed at 50°C. Overgo probes were designed using the Overgo Maker tool available for download through the Washington University Genome Sequencing Centre website, and used to screen the BAC library as previously described [22].

### Direct Sequencing

400 ng BAC DNA was used in a sequencing reaction containing 4 μl BigDye Terminator version 3.1 (Applied Biosystems) and 5 pmol primer. Cycling conditions for sequencing reactions were: 95°C for 5 minutes followed by 99 cycles of 96°C for 10 seconds, 50°C for 10 seconds and 60°C for 4 minutes. Reactions were precipitated and sent to AGRF for capillary separation.

### RNA isolation

Tissue was collected under The Australian National University Animal Experimentation Ethics Committee proposal numbers R.CG.08.03 and R.CG.11.06. RNA was isolated from tissue using the GenElute Mammalian Total RNA Miniprep Kit (Sigma-Aldrich, Sydney, Australia).

### Reverse transcription

First strand synthesis reactions were carried out using a random hexamer primer in the SuperScript III First-Strand Synthesis System for RT-PCR (Invitrogen, Australia). The integrity of the RNA and success of the first strand synthesis reaction was tested by PCR using the Quantum RNA 18S Internal Standards (Ambion). Control PCR amplification of 18S standards was carried out for all RNA samples. An 18S product was amplified from all first strand synthesis reactions (RT+), which confirmed the integrity of the RNA, and the quality of the first strand synthesis reaction. Control 18S products could not be amplified from any RNA samples on which a first strand synthesis reaction had not been performed (RT-), ensuring that there was no genomic DNA contamination of the RNA samples (results not shown). Control reactions were followed by RT-PCR amplification of marsupial MRX genes using gene-specific primers (Table 2).

### Fluorescence *in situ *hybridisation

Male tammar wallaby fibroblast cells were cultured and metaphase chromosome spreads prepared on glass slides as previously described [23]. BAC DNA was labelled by nick translation and hybridised to the chromosome preparations as previously described [17]. Fluorescence was visualised with a Zeiss Axioplan epifluorescence microscope fitted with a 100-W mercury lamp and a SPOT RT Monochrome CCD camera (Diagnostic Instruments Inc., Sterling Heights, MI, USA). IPLab imaging software (Scanalytics Inc., Fairfax, VA, USA) was used to capture and enhance images.

A localisation was confirmed by capture of images of at least 10 metaphase spreads on which a specific localisation could be observed on two X chromatids or at least three chromosome 5 chromatids.

## Authors' contributions

RJD and MLD carried out the library screening. DAM and RJD carried out the localisation experiments. DAM carried out the expression analysis. JED localised *TSPAN7*. MLD participated in the design and coordination of the study, and drafted and revised the manuscript. JAMG contributed to the design of the study, and preparation and revision of the manuscript. All authors read and approved the final and revised manuscript.
